# Longitudinally Extensive Transverse Myelitis in a Patient With Systemic Lupus Erythematosus: A Case Report and Literature Review

**DOI:** 10.7759/cureus.68337

**Published:** 2024-08-31

**Authors:** Prakash Banjade, Sudip Bastakoti, Ashmita Poudel, Munish Sharma

**Affiliations:** 1 Medicine, Manipal College of Medical Sciences, Pokhara, NPL; 2 Internal Medicine, Tribhuvan University Teaching Hospital, Institute of Medicine, Kathmandu, NPL; 3 Neurology, Tower Health, Pennsylvania, USA; 4 Pulmonary and Critical Care, Baylor Scott & White Medical Center - Temple, Temple, USA

**Keywords:** quadriparesis, neuromyelitis optica spectrum disorder, systemic lupus erythematosus, longitudinally extensive transverse myelitis, transverse myelitis

## Abstract

Longitudinally extensive transverse myelitis (LETM) is a rare but severe neurological complication of systemic lupus erythematosus (SLE). The existing literature contains only limited information about this condition. We present a case of a 38-year-old female with SLE who presented with quadriparesis. Magnetic resonance imaging (MRI) of the brain and spinal cord showed T2-weighted high signal intensity involving the brainstem, bilateral middle and inferior cerebellar peduncles, and C1-C7 spinal cord segments. Early intervention with high-dose methylprednisolone and cyclophosphamide was initiated, resulting in partial clinical recovery. A comprehensive literature review highlights the importance of early diagnosis and treatment, discusses the potential etiologies, and explores the prognostic factors influencing patient outcomes. This case report underscores the need for a high level of clinical suspicion and prompt therapeutic intervention to improve prognosis in SLE patients presenting with LETM.

## Introduction

Transverse myelitis (TM) encompasses diverse syndromes that cause acute or subacute spinal cord dysfunction, leading to motor, sensory, and autonomic disturbances below the lesion level [1]. Acute TM (ATM) usually refers to short-segment myelitis that typically involves lesions extending only one to two vertebral segments. In contrast, longitudinally extensive TM (LETM) is a rare and severe form affecting three or more contiguous vertebrae, as seen on T2-weighted MRI [2]. LETM is commonly associated with neuromyelitis optica (NMO), but it can also result from spinal cord infarction, postinfectious events, and autoimmune disorders like systemic lupus erythematosus (SLE) [3]. SLE can present with TM either at the onset or many years later [4]. Despite aggressive immunosuppressive treatment, half of LETM patients experience poor neurological outcomes, and more than a quarter suffer long-term disability [5]. In this paper, we discuss a rare instance of LETM in a patient with SLE and review the related literature.

## Case presentation

A 38-year-old female, with a past medical history of SLE diagnosed five years back, presented to our hospital with weakness of bilateral upper and lower limbs for the last four months. Weakness was symmetrical in onset and gradually progressive and was associated with a burning sensation in the upper extremities. The patient also complained of numbness in bilateral lower extremities throughout this time. She was taking prednisolone 30 mg daily, hydroxychloroquine 200 mg twice daily, and leflunomide 20 mg daily. On presentation to our hospital, her blood pressure was 120/70 mmHg, heart rate was 70 beats per minute, respiratory rate was 12 breaths per minute, and temperature was 98˚F. Neurological examination revealed power of 3/5 in bilateral upper limbs and 2/5 in bilateral lower limbs. Deep tendon reflexes showed generalized hyperreflexia with 3+ reflexes in bilateral upper limbs and bilateral knees with bilateral ill-sustained ankle clonus. Planters were bilateral and upgoing. Sensory system examination revealed no abnormalities. The cranial nerves examination did not show any abnormality. The rest of the system examination was normal.

A lumbar puncture was performed which showed clear fluid with a total count of 5/mm^3^ (all lymphocytes), glucose of 45 mg/dL, microprotein of 25 mg/dL, and adenosine deaminase of 6.4 U/L. A full summary of laboratory investigation results is shown in Table 1 and Table 2.

**Table 1:** Summary of serum laboratory results. BUN: Blood Urea Nitrogen, ALT: Alanine Amino Transferase, AST: Aspartate Amino Transferase, ESR: Erythrocyte Sedimentation Rate, CRP: C-Reactive Protein, MOG: Myelin Oligodendrocyte Glycoprotein, NMO: Neuromyelitis Optica, C3: Complement 3, dsDNA: Double-Stranded DNA, Anti-HCV: Antibodies Against Hepatitis C Virus, HbsAg: Antibodies Against Hepatitis B Surface Antigen

Lab Parameters	Patient value	Reference range
WBC (per mm^3^)	13,700	4000-11,000
Neutrophils (%)	82	40-75
Lymphocytes (%)	15	20-45
Eosinophils (%)	1	0-6
Monocytes (%)	2	2-10
Hemoglobin (g/dL)	10.6	12-16
Platelets (per mm^3^)	235,000	150,000-400,000
MCV (fl)	71.33	82-92
BUN (mmol/L)	5.3	2.1-8.5
Creatinine (microMol/L)	78	58-96
AST (U/L)	37	5-40
ALT (U/L)	39	5-45
ESR (mm/hr)	17	0-20
CRP (mg/L)	2	0-6
Vitamin B12 (pg/mL)	528	187-833
TSH (microIU/mL)	0.825	0.35-4.94
Anti-mog antibody	Negative	-
Anti-NMO antibody	Negative	-
C3 (mg/dL)	114	90-180
Anti-ds DNA (IU/mL)	25.1	< 30- negative, > 50 - positive
Anti-phospholipid antibody	Negative	-
Anti-HCV	Negative	-
VDRL	Non-reactive	-
HIV	Negative	-
HbsAg	Negative	-

**Table 2:** Summary of cerebrospinal fluid analysis results. ADA: Adenosine Deaminase, HSV-1: Herpes Simplex Virus 1, HSV-2: Herpes Simplex Virus 2, EBV: Epstein-Barr Virus, VCA: Viral Capsid Antigen, CMV: Cytomegalovirus

Lab Parameters	Patient value	Reference range
Total count (per mm^3^)	5 (all lymphocytes)	0-5
ADA (U/L)	6.4	< 10
Glucose (mg/dL)	45	70-140
Microprotein (mg/dL)	25	< 45
RBC	Nil	Nil
CSF antibodies		
HSV1 IgG/ IgM	Negative	-
HSV2 IgG/IgM	Negative	-
CMV IgG/IgM	Negative	-
EBV VCA IgG/IgM	Negative	-
CSF culture	No growth in 48 hours of incubation at 37˚C	-

**Figure 1:**
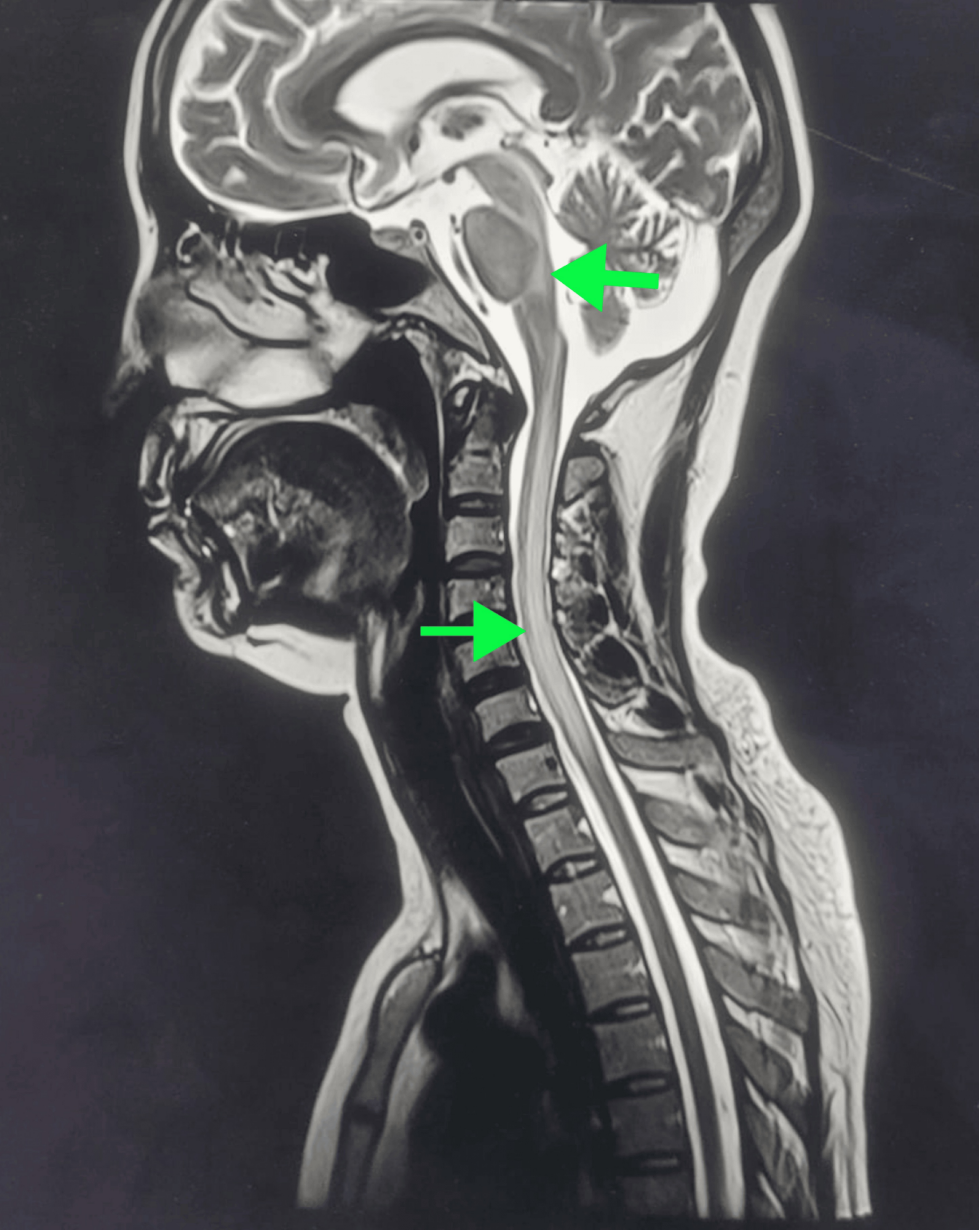
Sagittal T2-weighted MRI with gadolinium contrast showing high signal intensity in the brainstem and C1-C7 spinal cord segments.

**Figure 2:**
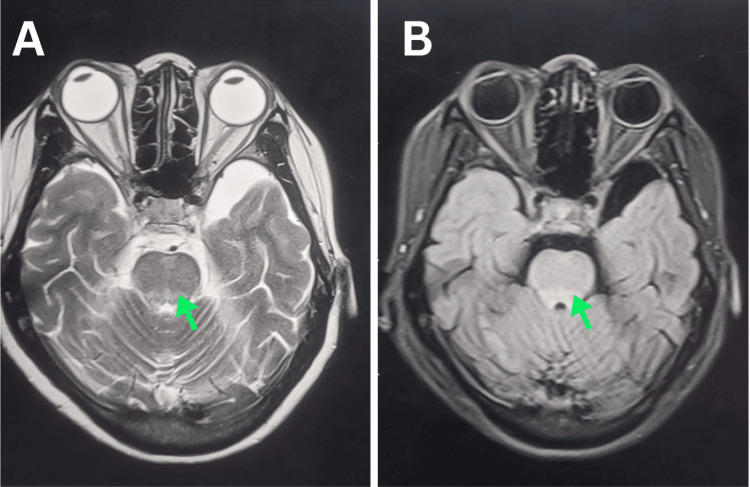
Axial T2 (A) and FLAIR (B) MRI brain showing high signal intensity at the level of the midbrain.

MRI of the brain and spinal cord with and without gadolinium contrast showed T2-weighted high signal intensity involving the brainstem, bilateral middle and inferior cerebellar peduncles, and C1-C7 spinal cord segments with mild cord expansion (Figures 1-2).

The initial differential diagnosis was neuromyelitis optica spectrum disorder (NMOSD) based on MRI findings, which was ruled out by a negative anti-NMO antibody. Based on the patient’s history, clinical presentation, and investigation findings, the diagnosis of longitudinally extending transverse myelitis as a complication of SLE was made. The patient was treated with 1 gram pulse methylprednisolone for five days and intravenous cyclophosphamide. The patient gradually achieved some clinical recovery and was discharged on the 10th day of hospital admission with planned to follow up in clinics for the next dose of cyclophosphamide.

## Discussion

TM is characterized by acute or subacute spinal cord dysfunction, resulting in paresis, a sensory level, and autonomic impairment below the level of the lesion [6]. LETM refers to complete or incomplete spinal cord dysfunction with a corresponding lesion on MRI that extends across three or more vertebral segments [2]. Demyelinating diseases, infections, and autoimmune inflammatory diseases such as SLE are the three leading causes of acute TM [7].

SLE is a chronic autoimmune condition that can impact any organ, including the nervous system. The term "neuropsychiatric SLE" (NPSLE) describes the direct and primary pathological involvement of neuroanatomy affected by SLE. It results primarily from the disease processes such as inflammation or thrombosis [8]. Studies indicate that about one-third to one-half of SLE patients experience neurological or neuropsychiatric symptoms [9]. The broad spectrum and heterogeneity of its phenotypes make diagnosis challenging for clinicians [10]. The American College of Rheumatology (ACR) classified it into 19 manifestations in 1999, categorizing them into two categories: (1) affecting the central nervous system (CNS) (which was again subdivided into focal and diffuse manifestations) and (2) affecting the peripheral nervous system [8]. TM is a focal manifestation of the CNS. The estimated prevalence of myelitis in SLE is around 2.1% [4]. ATM associated with SLE is a rare but severe complication, potentially causing significant neurological deficits [5].

A case series involving 14 patients with SLE and TM documented that TM was either the first manifestation or presented within five years of the diagnosis of SLE [4]. A retrospective study analyzing lupus myelitis cases showed that nearly two-thirds (61/94) were associated with active lupus, while one-third (33/94) were linked to low disease activity [11]. In our case, the C3 level was 114 mg/dL (reference range: 90-180 mg/dL), and the anti-dsDNA level was 25.1 IU/mL (reference: < 30 negative, >50 positive), indicating that the patient developed TM during a state of low disease activity in lupus (LLDAS).

Pathophysiology

The pathophysiology of NPSLE is not entirely understood. Vasculitis and small vessel thrombosis are believed to be the two main processes responsible for causing axonal damage through ischemia and necrosis. Thoracic level involvement is common due to the smaller caliber vessels in the medullary vasculature, making them more susceptible to thrombosis. If antiphospholipid antibodies (aPL) are present in the serum, this could suggest that thrombosis plays a predominant pathogenic role [12]. A case series by Katsiari et al. found that, in patients with TM due to NPSLE, the association with aPL occurs with a frequency of 50-100%. These antibodies suggest a higher risk of neurological manifestations in patients with SLE [13].

Clinical presentation

The prevalence of SLE-related LETM is higher in young women, with 77% of patients being female and an average age of 30 years, and it may be the initial presenting feature of SLE [14]. TM usually has an acute to subacute onset, with neurological deficits peaking within a few weeks. Acute complete TM (ACTM) is characterized by paresis/plegia, sensory dysfunction such as numbness or paresthesias with a sensory level, and autonomic disturbance below the lesion. Acute partial TM (APTM) leads to asymmetric clinical features in specific anatomic tracts. It may present as various spinal cord syndromes such as hemi-cord (Brown-Sequard), central cord, posterior column syndrome, and selective tract impairment. APTM presents as either motor or sensory dysfunction, but not both [6].

In the acute phase, spinal shock syndrome may manifest, resulting in reduced or absent limb tone and muscle stretch reflexes. This can lead to diagnostic confusion with Guillain-Barre syndrome (GBS). Spinal shock can last from days to weeks, with an average duration of four to six weeks after an insult. Over time, characteristic symptoms of upper motor neuron (UMN) syndrome-spasticity, hyperreflexia, and extensor plantar responses become evident [6,15].

Patients with LETM form a distinct subgroup of TM, different from those with shorter lesions. They have a low risk of developing multiple sclerosis (MS) but experience more severe clinical symptoms [2]. Despite aggressive immune suppression, half of LETM patients have unfavorable neurological outcomes, and over a quarter suffer from long-term disability [5].

Other symptoms of SLE, such as Joint pains, myalgias, morning stiffness, and integumentary manifestations, may be present in patients with active disease states.

Evaluation

History: The initial step involves assessing whether the condition is likely a myelopathy. Symptoms that should prompt a clinician to consider myelopathy include weakness and sensory impairment in a myelopathic distribution, along with bowel or bladder dysfunction [6]. It is essential to consider the patient's medical history for factors that may cause myelopathy, such as autoimmune disorders, systemic illnesses, previous use of medications such as methotrexate, tumor necrosis factor-alpha inhibitors, exposure to radiotherapy, immune checkpoint inhibitors, and substance abuse [16].

Examination

During an examination, it is crucial to identify the site of neurological dysfunction. The presence of motor and/or sensory dysfunction without affecting the head or face gives suspicion of spinal cord lesions. When a patient has a reported or observed sensory level, the possibility of myelopathy is considered until proven otherwise. CNS motor deficits present as weakness and long tract signs, such as spasticity, increased reflexes, and upgoing planters. A neuro-ophthalmological assessment is necessary for ophthalmic manifestations such as optic neuritis, which can offer important diagnostic insights.

Urgent Neuroimaging

Rapid neuroimaging of the entire spinal cord is crucial to rule out compression. Gadolinium-enhanced MRI is the preferred diagnostic tool for confirming myelitis, including cases caused by lupus. It also helps exclude other spinal cord issues, such as bruises or tumors. Longitudinal spinal cord compromise is a more common imaging finding than the transverse one [6,17]. In patients with LETM, MRI shows T2-weighted hyperintense lesions that span three or more vertebral segments [2]. For patients with hyperacute (<12 hours) or acute onset of myelopathic symptoms, diffusion-weighted imaging (DWI) of the spine should be performed to check for spinal cord infarction [18].

All patients should undergo a brain MRI with and without gadolinium to identify any concurrent or previous lesions that could offer insights into the cause and help differentiate various CNS demyelinating disorders. In patients with partial TM, the presence of brain lesions similar to those seen in MS indicates an 80% risk of progressing to clinically definite MS within three to five years [19].

*Cerebrospinal Fluid *(*CSF) Analysis*

Approximately one-half of patients with LETM have CSF abnormalities. CSF pleocytosis is expected as moderate lymphocytosis, typically with a cell count below 100/microL. Some patients can have mild or moderately low (usually >30 mg/dL) CSF glucose. However, a normal CSF glucose does not exclude the diagnosis [17].

Electrophysiologic tests may be useful in assessing TM patients [6]. In cases of SLE-associated TM, electromyography and nerve conduction studies (EMG/NCS) may reveal anterior horn cell loss at the affected levels and suprasegmental weakness. However, these tests are typically normal unless myeloradiculitis is present.

Serologic Tests and Autoantibodies

To identify treatable causes of myelopathy, serum vitamin B12 levels, thyroid function tests, and serologies for syphilis and HIV should always be conducted. Vitamin E, serum copper, and ceruloplasmin levels should also be checked in individuals at risk of deficiency. All patients with TM should be tested for serum aquaporin-4-specific autoantibodies (NMO-IgG) and anti-MOG antibodies to rule out NMOSD and MOG antibody-associated demyelinating disease (MOGAD), respectively, as these may also present as TM, including longitudinally extending TM [20,21].

Table 3 summarizes the investigation needed in patients with TM [6,17,20,21].

**Table 3:** Summary of investigations required in patients with transverse myelitis. Source: Refs. [6,17,20,21]

In all patients suspected of TM	Additional tests if LETM is suspected	May require
MRI of the spine	Serum NMO-IgG	Paraneoplastic panel
Brain MRI	Serum anti-MOG antibodies	Infectious serology
Cerebrospinal fluid analysis	Serum ESR, C-RP, ANA, rheumatoid factor, antiphospholipid antibodies, ANCA	CSF culture
Serum: B12, methylmalonic acid, HIV antibodies, syphilis serology, thyroid stimulating hormone (TSH), Free T4, 25-hydroxyvitamin D	Visual-evoked potentials	Nerve conduction studies and electromyography

Differential diagnosis: Some SLE patients with myelitis test positive for anti-aquaporin-4 antibodies, indicating the presence of comorbid NMOSD. NMO is an immune-mediated inflammatory demyelinating disorder of the CNS, characterized by recurring episodes of LETM and optic neuritis LETM, frequently accompanied by other autoimmune diseases [2,22]. Other patients may have positive MOG autoantibodies, which could signify comorbid MOGAD [23]. Although MS and SLE are not as strongly associated as some other autoimmune disease pairings, comorbid MS should also be taken into account. In one population-based study conducted in the United Kingdom, the elevated incident rate ratio (IRR) for MS in those with SLE was nearly twice as high as the risk in the general population [24]. In patients with SLE, myelitis can also result from infections due to their immunosuppressed condition, so a thorough CSF evaluation is essential to identify the cause. Other potential diagnoses include spinal cord infarction, which typically occurs suddenly, similar to a stroke, and may be associated with antiphospholipid seropositivity [25].

Treatment

Acute Treatment

The European League Against Rheumatism (EULAR) guidelines for managing neuropsychiatric symptoms in lupus advise the prompt use of methylprednisolone and intravenous (IV) cyclophosphamide. The recommended regimen includes 1-gram IV pulses of methylprednisolone for three days, followed by prednisone at 1 mg/kg/day starting the fourth day, with a gradual taper over one to three months. Additionally, IV cyclophosphamide should be administered at 0.75-1 g/m² of body surface area [26]. In some cases, especially when concomitant NMOSD causes myelitis, plasma exchange is also administered concurrently to patients who are severely affected or who do not respond to glucocorticoids. Intravenous gammaglobulin may be utilized either as an initial treatment or, in cases that are resistant to other therapies, either alone or alongside standard treatments [27]. Observational studies have indicated positive results when patients with TM and SLE, who do not have comorbid NMOSD, are treated with high-dose glucocorticoid pulses followed by a tapering course of oral steroids, often in conjunction with cyclophosphamide [28,4,29].

Long-Term Treatment

Cyclophosphamide is usually administered for three to six months, followed by transitioning to a less toxic drug, including mycophenolate, azathioprine, or rituximab for maintenance therapy. This regimen effectively manages SLE disease activity and lowers the recurrence risk [30,31]. Considering the medication's risk, tolerability, and overall effectiveness in managing SLE disease activity, immunosuppressive treatment is usually maintained for years in order to lower the risk of CNS recurrence.

Prognosis

Patients with LETM often have a poor prognosis. A systematic review by Espinosa et al. indicated that most patients had residual neurologic deficits or no clinical recovery at all; only 14% recovered completely [32]. A case series indicated that early treatment significantly improves outcomes for TM related to SLE, with the best results seen when treatment starts within one week; efficacy is considerably lower after this period [33]. Prognosis is associated with the rate of emergence of neurologic symptoms. The prognosis is worst in cases of hyperacute presentations, which usually develop over several hours [34]. Some reports suggested a correlation between antiphospholipid antibodies and poor outcomes in ATM and reported that patients with LETM testing positive for aPL antibody may have better clinical outcomes if treated with anticoagulation or antiplatelet therapy [35]. However, a recent systematic review of cases of SLE with acute TM and LETM did not support these findings [13]. Our patient had a less acute onset of neurologic symptoms, tested negative for aPL antibodies, and received prompt therapeutic interventions after diagnosis, which led to good clinical outcomes.

## Conclusions

LETM is a severe manifestation of SLE, often resulting in poor clinical outcomes. Despite the use of aggressive immunosuppressive therapies, about half of LETM patients experience significant neurological deficits, and more than a quarter endure long-term disabilities. Early detection and treatment are critical for better outcomes; therefore, any acute, widespread neurological decline involving motor disturbances, sensory level, and autonomic dysfunction in lupus patients should prompt consideration of LETM. It is also important to exclude other causes of LETM and any concurrent autoimmune diseases. Early administration of high-dose methylprednisolone and cyclophosphamide can improve clinical outcomes.
